# Microbiological and microstructural analysis of indwelling bladder catheters and urinary tract infection prevention

**DOI:** 10.1590/1980-220X-REEUSP-2021-0552

**Published:** 2022-04-01

**Authors:** Mateus Flávio Sousa, Luís Guilherme Oliveira Reis, Valéria da Silva Baracho, Sara Luísa de Oliveira, Gessiane de Fátima Gomes, Thabata Coaglio Lucas

**Affiliations:** 1 Universidade Federal dos Vales do Jequitinhonha e Mucuri, Diamantina, MG, Brazil.; 2 Santa Casa de Caridade de Diamantina, Diamantina, MG, Brazil.; 3 Universidade Federal dos Vales do Jequitinhonha e Mucuri, Programa de Pós-Graduação em Ciências da Saúde, Diamantina, MG, Brazil.; 4 Universidade Federal dos Vales do Jequitinhonha e Mucuri, Programa de Pós-Graduação em Ensino em Saúde, Diamantina, MG, Brazil.

**Keywords:** Urinary Tract Infection, Urinary Catheters, Microscopy, Electron, Scanning, Public Health, Public Health Surveillance, Infecciones Urinarias, Catéteres Urinarios, Microscopía Electrónica de Rastreo, Salud Pública, Vigilancia en Salud Pública, Infecções Urinárias, Cateteres Urinários, Microscopia Eletrônica de Varredura, Saúde Pública, Vigilância em Saúde Pública

## Abstract

**Objective:**

To analyze the microbiological and microstructural part of indwelling urinary catheters and their association with urinary tract infection prevention.

**Method:**

This is a cross-sectional study, from June to December 2020, in which 42 indwelling urinary catheter tips and sterile urine samples were collected for analysis of crystals in optical microscopy and biofilms in scanning electron microscopy. Culture analysis and specification of the type of bacteria were performed.

**Results:**

It was found that 35.71% of the samples had mature biofilm adhered to the catheter tip. Biofilms of *Proteus mirabilis*, *Enterococcus faecalis*, *Staphylococcus epidermidis*, *Enterococcus faecium* and *Enterobacter cloacae* stood out. The presence of magnesium-ammonium-phosphate crystal was associated with the presence of urinary tract infection and with *Proteus mirabilis*. There was a significant association (p = 0.001) between the use of prophylactic antibiotics versus urine culture >10^5^ CFU/mL.

**Conclusion:**

The analyzes contributed to clinical practice, as it reinforces the development of effective and monitored strategies on cultures and urinary tract infection prevention associated with indwelling urinary catheters.

## RESUMO

**Objetivo:**

Analisar a parte microbiológica e microestrutural dos cateteres vesicais de demora e sua associação com a prevenção de infecção do trato urinário.

**Método:**

Transversal, entre junho e dezembro de 2020, em que foram coletadas 42 pontas cateteres vesicais de demora e amostras de urina estéril para análise de cristais em microscopia óptica e de biofilmes em microscopia eletrônica de varredura. Fez-se analise de cultura e especificação do tipo de bactérias.

**Resultados:**

Verificou-se que 35,71% das amostras apresentaram o biofilme maduro aderido à ponta do cateter. Destacaram-se biofilmes de *Proteus mirabilis*, *Enterococcus faecalis, Staphylococcus epidermidis*, *Enterococcus faecium* e *Enterobacter cloacae*. A presença do cristal magnésio-amônio-fosfato foi associada à presença de infecção do trato urinário e ao *Proteus mirabilis.* Verificou-se associação significativa (p = 0,001) entre o uso de antibiótico profilático *versus* a urocultura >10^5^ UFC/mL.

**Conclusão:**

As análises contribuíram para a prática clínica, pois reforçam a elaboração de estratégias efetivas e monitoradas sobre culturas e prevenção de infecção do trato urinário associada ao cateter vesical de demora.

## RESUMEN

**Objetivo:**

Analizar la parte microbiológica y microestructural de los catéteres urinarios permanentes y su asociación con la prevención de la infección del tracto urinario.

**Método:**

Transversal, de junio a diciembre de 2020, en el que se recolectaron 42 puntas de sonda urinaria permanente y muestras de orina estéril para análisis de cristales en microscopía óptica y biopelículas en microscopía electrónica de barrido. Se realizaron análisis de cultivo y especificación del tipo de bacteria.

**Resultados:**

Se encontró que el 35,71% de las muestras presentaban biopelícula madura adherida a la punta del catéter. Se destacaron las biopelículas de *Proteus mirabilis, Enterococcus faecalis, Staphylococcus epidermidis, Enterococcus faecium* y *Enterobacter cloacae*. La presencia de cristales de fosfato de magnesio-amonio se asoció con la presencia de infección del tracto urinario y con *Proteus mirabilis.* Hubo una asociación significativa (p = 0,001) entre el uso de antibióticos profilácticos versus urocultivo >10^5^ UFC/mL.

**Conclusión:**

Los análisis contribuyeron a la práctica clínica, ya que refuerzan el desarrollo de estrategias efectivas y monitoreadas sobre cultivos y prevención de la infección del tracto urinario asociada a los catéteres urinarios permanentes.

## INTRODUCTION

Urinary tract infection (UTI) is one of the major healthcare-associated infections (HAI). In the USA, about 80% of UTIs are associated with indwelling bladder catheter (IBD) (CA-UTI) and approximately 15% occur due to inadequate indications^(1)^. In Brazil, CA-UTI are responsible for 35 to 45% of infections acquired in hospitals^(2)^. CA-UTI may be associated, mainly, with inadequate aseptic handling, prolonged catheter use, inadequate indication and bacterial colonization due to the formation of biofilms^(3)^.

Biofilms can promote IBD blockage, adhesion via strong covalent bonds between the exopolysaccharide matrix (EPS) and the polymeric device wall, consequently leading to polycolonic formation with co-colonization of different types of bacteria adhered to the catheter wall^(4–5)^. An experimental study indicated that, regardless of the type of polymeric material of IBD, there was growth of viable bacteria, such as *Escherichia coli, Pseudomonas aeruginosa* and *Proteus mirabilis*, for a period ranging from 6 to 72 hours post-insertion, adhered to the EPS matrix on the device wall^(5)^. After 72 hours, signs of biofilm detachment and the presence of new areas of recolonization were observed under microscopy. In clinical practice, in addition to CA-UTI, it can lead to the formation of fouling and occlusions of catheters, favoring the precipitation of calcium, magnesium, ammonium and phosphate crystals, causing urinary stasis in the bladder or delaying emptying, contributing to increased proliferation of microorganisms^(6)^.

Although studies on biofilm^(6,7,8,9)^ that develop on the IBD wall show remarkable progress in science, truly effective and widely applicable strategies to manage biofilm-related complications faced by catheterized patients are still a gap in scientific knowledge and a challenge for clinical practice.

In another study, the authors assessed, by culture, biofilm detection tests, biochemical identification of bacteria, and antibiotic sensitivity tests of UTI pathogens in patients with IBD^(8)^. *Escherichia coli, Klebsiella pneumoniae* and *Pseudomonas aeruginosa* were identified as the main multidrug-resistant bacteria that may be linked to biofilms^(8)^. Other bacteria, such as *Enterococcus fecalis,* to *Stahphiloccus aureus* and *Proteus mirabilis,* also showed resistance, but to a limited spectrum of antibiotics. On the other hand, all of them were identified as biofilms on the IBD wall^(8)^.

In this sense, scientific evidence reinforces the need to adopt effective measures to prevent CA-UTI, especially during the device insertion and maintenance, aiming at patient safety and clearly indicating to health professionals, the ease of infection and dispersion of bacteria in the catheter from the moment it is inserted and maintained in patients. Catheterization can lead to serious complications, such as pyelonephritis, bacteremia, septicemia, and sepsis, which are responsible for increased morbidity and mortality in patients with IBD^(6–9)^. Thus, the formation of biofilms, fouling and the emergence of antibiotic-resistant microorganisms are still the main challenges in CA-UTI management, and must be actively monitored, so that public health policies can be taken to guide, not only health professionals, but also patients, in order to obtain effective surveillance in managing IBD-related adverse events.

Faced with uncertainties and gaps both in clinical practice and in scientific knowledge regarding UTI prevention associated with IBD, the question is: how can the microstructural and microbiological analysis of IBD be associated with UTI prevention?

## METHOD

### Design of Study

This is a cross-sectional observational study. The present study used the STROBE protocol for cross-sectional studies.

### Local

Neurological, surgical, medical clinic and intensive care center in a philanthropic health institution in the countryside of Minas Gerais.

### Selection Criteria

Participants older than 18 years who were using IBD for more than 48 hours and patients without a diagnosis of UTI at admission were included. Unconscious patients and those with neurogenic bladder, urethral stenosis, urinary incontinence, bladder dysfunction and prostate hyperplasia were excluded.

### Sample

Participants were selected, by convenience sample, and 42 samples were collected between June and December 2020, considering the statistically recommended minimum number of 30 individuals^(10)^.

It is noteworthy that, although there was a sample larger than the minimum necessary for statistical analysis, due to the COVID-19 pandemic in 2020 and changes in the institution’s protocol, especially not authorizing the presence of researchers in the sectors, the sample was concluded in 42 participants.

### Data Collection

#### Stage 1: Material collection and analysis for urine culture and catheter tip

For urine culture, immediately before IBD removal, about 10 mL of sterile urine was collected in a sterile collection flask. The sample was collected after a medical prescription that there was no longer indication and IBD use.

Aseptically, 5 cm from the IBD tip was also removed. This was cut with sterile scissors to divide the tip into two parts: a) one part for culture, being inserted in a sterile collector with 0.9% saline solution; b) another part being inserted in a sterile collector, which contained 70% alcoholic solution, being used for visualization in scanning electron microscopy (SEM). This tip was conserved at 4^°^C until observation. The IBD segment, stored in 0.9% saline solution, was analyzed up to a maximum of one hour after collection.

The samples were transported to the Bioprocess laboratory of the Department of Pharmacy of the *Universidade Federal dos Vales do Jequitinhonha e Mucuri* (UFVJM), to perform the culture and isolate the bacteria found, using the gram staining method to identify gram-positive and gram-negative bacteria.

For culture, both in the urine sample and in the IBD tip, the surface scattering technique was performed. Müeller-Hinton agar supplemented with 5% sheep blood and a differential rich medium (Cled BD agar) were used to analyze growth, quantification and isolation. After sown, samples were incubated for 24 hours at 37°C.

After that, colony counting was performed manually with the counting of the total load (CFU/mL). Isolation was performed with the aid of a sterile inoculation loop, in which different types of colonies were delicately collected, if this was the case for that sample. Each isolated colony was deposited in different Eppendorf microtubes, properly identified, containing 0.9% saline solution and 2 drops of sterile glycerol conserved at +4^°^C until the time of gram analysis.

After identification of gram+ and gram− bacteria, colonies were inserted and inoculated into Microscan Walkaway automated equipment (Siemens-Sacramento-CA-USA). Through this method, it was possible to identify the different types of bacteria and fungi that could have grown in the sterile sample from both IBD tip culture and urine.

For the catheter tip, the same method was also used; however, the tip was cultured on CLED agar by the scattering technique, and a second IBD fragment was also cultured in a medium containing a broth BBL Trypticase Soy Broth-BD^®^, vortexed for 1 minute. Next, 1 μL was sowed on Müeller-Hinton agar supplemented with 5% sheep blood and a differential rich medium (Cled BD agar), being incubated for 24 hours. After 24 hours, the colonies were counted and the strains were identified by the automated method mentioned above.

As a cut-off criterion of CA-UTI, patients undergoing catheterization for more than 48 hours or after catheter removal within 48 hours and who had fever (≥38^°^C), suprapubic tenderness, costovertebral angle pain or tenderness, when there was no other recognized cause, and positive urine culture with a maximum of two species of microorganisms with growth ≥10^5^ units of colony formation per mL of urine (CFU/mL) were standardized^(11)^. To assess the presence of pain, a professional nurse, part of this research, carried out a physical assessment with the patient after the IBD removal, checking on fever.

#### Stage 2: Crystalline fraction collection and analysis

For analysis of urine crystals, 5 mL of urine was inserted into Falcon tubes, centrifuging at 1,500 revolutions per minute (RPM) for 5 min at a temperature of 20^°^C. The urine sediments were observed under optical microscopy (40x) with the aid of a Neubauer camera for counting and typology of the crystals. Crystal analysis aimed to compare the presence of specific crystals that could be associated with CA-UTI.

#### Stage 3: Scanning electron microscopy analysis

In SEM, the degree of biofilm development and maturation was observed. The IBD segments were cut longitudinally, about 1 cm, to expose both the endoluminal and external surfaces. The cut was performed under a biological safety cabinet using a sterile scalpel. The sample was delicately manipulated, so that it did not allow the destruction of biofilm, microorganisms or crystalline precipitates that were visualized.

After removing the samples of 70% alcohol, they were washed with sterile water and dehydrated, in ascending order, with alcoholic solutions: 70% alcohol for 10 minutes, 90% alcohol for 10 minutes and 100% ethanol for 10 minutes. Next, the sample remained in the biosafety cabinet for 15 minutes for drying.

Scanning electron microscope (XL 30 ESEM FEG, Fei-Philips, The Netherlands) was used. The samples were placed on a metal support, and then gold coating (Sputter Coater K500X, Emitech.), with 15 mA, was made for 2.5 minutes. High-definition micrographs were obtained with a magnification of 500 to 4,000x.

#### Stage 4: Data collection in medical records

In participants’ medical records, the following variables were collected: sex; age; patient diagnosis; risk factors (previous UTI, diabetes mellitus, systemic arterial hypertension, immunosuppressed, associated urological pathology, presence of urinary stent and history of urological surgery); and urine culture. Data collection in the medical records occurred from June to December 2020.

### Data Analysis

For descriptive analysis, mean ± standard deviation and minimum and maximum values were used. The Shapiro-Wilk test was used to analyze the normality of the quantitative variables. For analysis of means, Student’s t-test was performed. For the comparison of three groups, the ANOVA with Tukey post hoc (parametric) was used. Pearson’s and Fisher’s chi-square test was used to analyze the categorical variables. The level of 0.05 was used in a fixed manner.

### Ethical Aspects

This study was approved by the Institutional Review Board (IRB) of UFVJM, under Opinion 3,523,335. This study was approved in 2019 and is in accordance with Resolution 466/12 of the Brazilian National Health Council (*Conselho Nacional de Saúde*). The participants who agreed to take part in the study signed the Informed Consent Form (ICF).

## RESULTS

**Chart 1:**
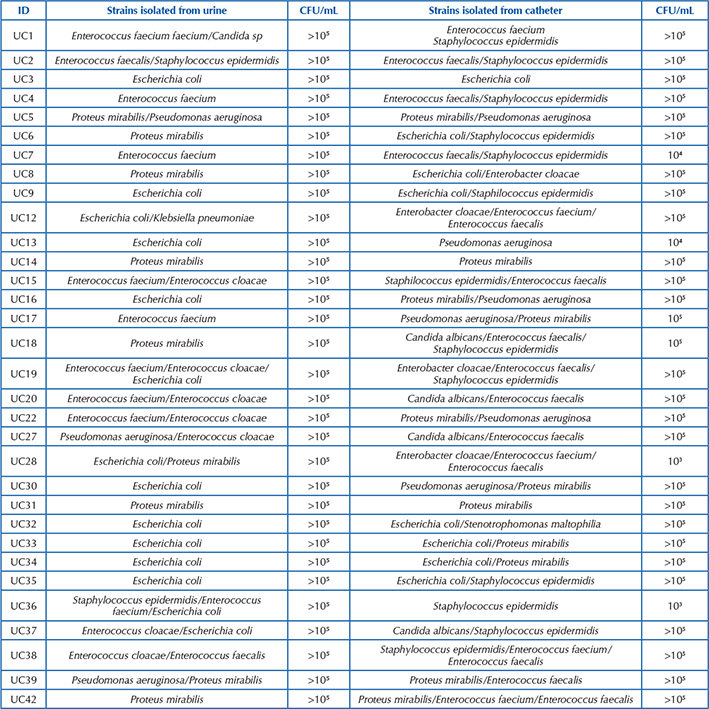
Comparison of the results of participants’ urine culture and indwelling urinary catheter tip culture – Diamantina, MG, Brazil, 2020 (N = 42).

Chart 1 presents the results of urine culture and IBD tip culture of the 42 patients in whom the samples were collected.

It was found that, of the 42 samples collected, 10 (23.80%) had no growth either in the urine culture or in IBC tip culture.

As for urine culture, there were results in the medical records of only 8 patients. UC8: *proteus sp*, UC9: *Escherichia coli*, UC15: *Enterococcus faecium/Enterococcus cloacae*, UC19: *Enterococcus faecium/Escherichia coli*, UC28: *Proteus sp/Escherichia coli*, UC32 and UC36: *Escherichia coli* and UC37: *Enterococcus cloacae/Escherichia coli*. All had >10^5^CFU/mL. However, the signs and symptoms of UTI in these patients had not been described.

*Identification of participants in codes. The other identifications did not grow either in catheter tip culture or in urine culture.

**Chart 2:**
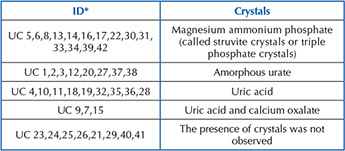
Detection of crystals identified in optical microscopy (40x) associated with participants – Diamantina, MG, Brazil, 2020 (N = 42).

It was found that 14 (33.3%) samples showed magnesium- ammonium-phosphate in the urine when viewed under optical microscopy; in 8 (19.04%), no crystals were observed; in 9 (21.42%), uric acid was observed; in 8 (19.04%), amorphous urate was observed; and in 3 (7.14%), uric acid and calcium oxalate were observed. Chart 2 presents the results of the types of crystals found in the study participants’ urine.

*ID: identification of participants in codes.

**Figure 1.**
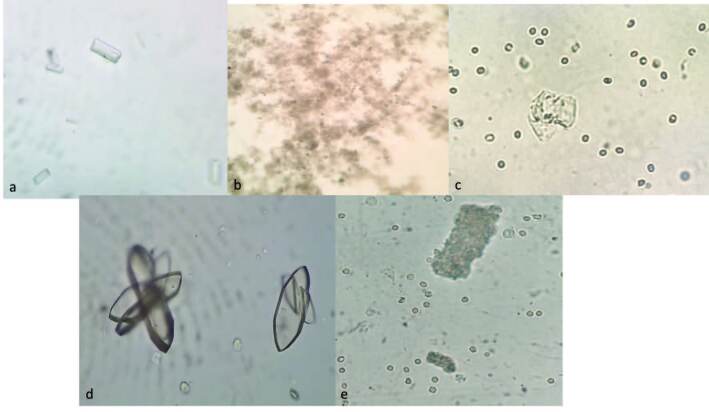
Urinary crystals visualized by optical microscopy. (a) magnesium-ammonium-phosphate crystals; (b) amorphous urate; (c) calcium oxalate (d) uric acid; (e) cylinder and erythrocytes.

Comparing Chart 1 with Chart 2, it was found that all samples in which magnesium-ammonium-phosphate was identified were those with urine culture and catheter tip culture that presented values ≥10^5^ CFU. Figure 1 presents magnesium- ammonium-phosphate crystals, amorphous urate, calcium oxalate, uric acid and cylinders with erythrocytes also identified in optical microscopy.

Of the 42 IBC tips analyzed, 15 (35.71%) presented mature biofilm at the IBC tip, namely: UC 4, 5, 9,12, 14, 15, 16, 19, 22, 30, 34, 37, 38, 39 and 42. All these patients also had suprapubic sensitivity and pain costovertebral angle soon after IBC removal.

**Figure 2.**
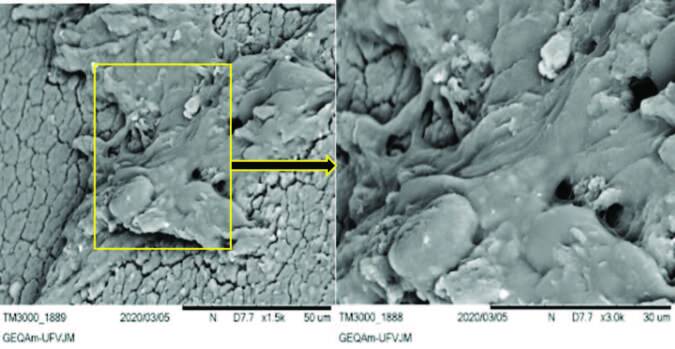
Mature biofilm adhered to the indwelling bladder catheter wall followed by magnification of the micrograph visualized in scanning electron microscopy.

Of the 15 samples, 9 (60.00%) were biofilms of *Proteus mirabilis*, and the others formed by *Enterococcus faecalis* (46.66%)*, Staphylococcus epidermidis* (33.33%)*, Enterococcus faecium* (13.33%), and *Enterobacter cloacae* (13.33%). Of the 42 samples, in 17 (40.47%) there was EPS production adhered to the IBC wall. Moreover, of the total of samples, in 10 (23.80%) there was a conditioning phase of cell adhesion to the surface, with adhered cocci and presence of protein residues. Figure 2 shows the characteristic of a mature biofilm with the presence of EPS adhered to the catheter wall. The presence of host fibrin aggregates in the biofilm matrix is verified.

**Figure 3.**
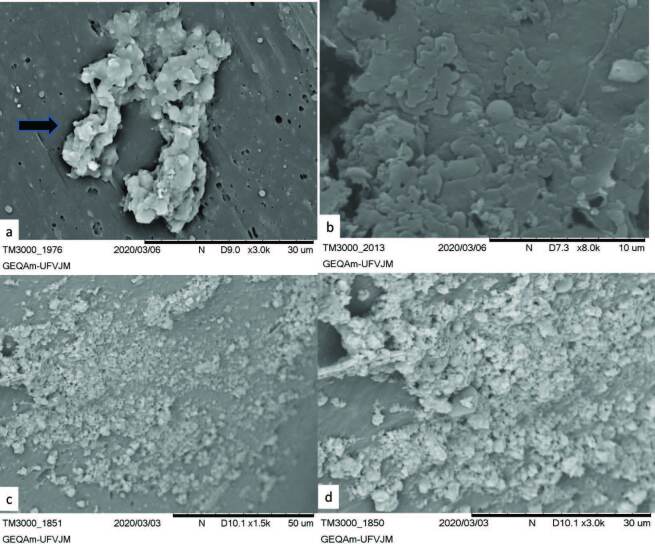
Production of exopolysaccharide matrix, cocci, and protein residues adhered to indwelling catheter visualized in scanning electron microscopy. (a) exopolysaccharide matrix production on the IBD wall and presence of cocci adhered to the biofilm; (b) exopolysaccharide matrix and cocci; (c) protein residue (phase of adhesion to the wall and exopolysaccharide matrix); (d) exopolysaccharide matrix enlargement.

Figure 3 presents micrographs that illustrate the EPS matrix on the IBD wall and the presence of cocci adhered to the biofilm. The presence of protein residue (adhesion phase to the wall and EPS matrix) adhered to the IBD wall was also verified.

As for the data that were taken from the medical records, the mean IBD length of stay was 9.43 ± 5.68 days, with a maximum time of 24 days and a minimum of 4 days. There was no significant association (p = 0.236) between length of stay and positive urine culture (>10^5^ CFU/mL). The IBD model was the two-way Foley for all participants. Regarding the type of IBD material collected, 35 (83.30%) were latex and 7 (16.70%) were silicone coated latex. There was no significant association (p > 0.05) between the type of IBD and presenting or not mature biofilm when observed in SEM.

The mean age was 56.36 years ± 15.76. As for sex, 17 (40.47%) were female and 25 (59.53%) were male.

Regarding the risk factors for CA-UTI, 16 (38.09%) did not have any risk factor, however, all of them had positive urine culture and catheter tip culture. Thus, having or not a risk factor for CA-UTI was not significantly associated (p > 0.05) in this study with having or not urine culture >10^5^ CFU. However, 6 (14.28%) of participants who had previous UTI, 6 (14.28%) who had chronic kidney disease and 3 (7.14%) who had both previous UTI and chronic kidney disease had catheter tip and urine culture values greater than 10^5^ CFU/mL. As for the other participants, 7 (16.66%) were diabetic and 4 (9.52%) were alcoholics. Of the participants who did not have bacterial growth, 6 (60%) were diabetic and 4 (40%) were alcoholics.

Regarding the use of antibiotics, it was found that 11 (26.19%) patients used therapeutic antibiotics due to their own diagnoses of the disease recorded in the medical records, such as ruptured ectopic pregnancy, sepsis of pulmonary and abdominal focus, septic shock and bacterial endocarditis, pelviperitonitis, spondylodiscitis and osteomyelitis. Of these, 1 (9.09%) sample presented urine culture and catheter tip culture >10^5^ CFU/mL. Of these, 11.8 (72.72%) had already started antibiotic therapy prior to the date of registration verified in the medical record.

Of the total participants, 17 (40.47%) used prophylactic antibiotics and 14 (33.33%) did not use antibiotics. Among those who did not use antibiotics during hospitalization, 10 (85.71%) had urine culture and catheter tip >10^5^ CFU/mL.

Of those participants who used prophylactic antibiotics, due to their own condition of hospitalization, and use of invasive procedures, 15 (88.23%) had urine culture >10^5^ CFU/mL. Of these, 11 had a catheter tip culture >10^5^ CFU/mL. Furthermore, patients used prophylactic antibiotics without previous urine culture. When antibiotic use (yes or no) was associated with urine culture >10^5^ CFU/mL, there was a significant association (p = 0.001).

## DISCUSSION

According to the results, it was found that, even though urine was collected in a sterile way, there was growth of microorganisms both in urine culture and in IBD tip culture (Charts 1 and 2). The microorganisms identified in sterile urine corroborate those most commonly associated with CA-UTI such as *Escherichia coli, Pseudomonas aeruginosa, Staphylococcus spp, Proteaus mirabilis, Klebsiella pneumoniae, Proteus vulgaris, Enterococcus spp* and *Candida albicans* ^(12)^.

In a study conducted in the United Kingdom, the authors collected 58 IBD from patients admitted to a health institution to verify the presence of bacteria in the catheter balloon and lumen^(13)^. The most prevalent microorganisms and common to the two selected sites in the IBD were *Escherichia coli* and *Enterococcus faecalis* ^(13)^. In this study, there was also a high prevalence at the IBD tip in *Enterococcus faecalis* 12 (28.57%) and *Escherichia coli* 8 (19.04%). Despite a lower prevalence, the presence of *Proteus mirabilis, Pseudomonas aeruginosa, Candida albicans, Staphylococcus epidermidis* and *Enterococcus faecium* ^(13)^ was also verified when compared to the present study.

In this study, the number of colonies identified was not statistically associated (p > 0.05) with the IBD length of stay. It is noteworthy that, even in a short IBD stay (<28 days), it was possible to visualize mature biofilms, which suggests a reflection on the probable risk in long-term patients (>28 days), especially in nursing homes, which can lead to detachment of biofilm cells.

Another factor that did not influence colony growth was the use of antibiotics. Most patients on prophylactic antibiotics presented urine culture values >10^5^ CFU. These results support an experimental study in which the authors did not verify an association between length of stay and bacteria growth, and they also verified that antibiotics did not significantly reduce (p > 0.05) the colonization in the lumens of IBD removed from patients^(13)^. It can be inferred that the inappropriate use of prophylactic antibiotics induces a response of microbial populations that accelerate the natural process of bacterial resistance. Another fact that drew attention is that patients used antibiotics prior to urine culture, which hinders the growth of microorganisms and provides false-negative results.

Also, mechanisms of bacterial resistance expression such as production of inactivating enzymes, alteration of the receptor for drug action, alternative metabolic pathways and even the formation of biofilms accelerate the maintenance process of an infectious process such as CA-UTI. In this study, it was possible to observe several stages of biofilm development (Figures 2 and 3), going from cell adhesion to the IBD surface to colony growth and extracellular medium maturation. As the biofilm growth medium in the IBD may contain blood, in addition to urine and different types of inorganic salts and crystals, the extracellular matrix may contain leukocytes, red blood cells, platelets, and fibrins, contributing to a specific type of mature biofilm that may be susceptible to cell breakdown and detachment from the biofilm matrix.

Another factor that may contribute to a specificity of the type of mature biofilm visualized in this study was that 60% were formed by *Proteus mirabilis*, which, in turn, develops a crystalline biofilm that encrusts on the IBD surface and blocks urinary flow^(14)^ (Chart 2 and Figure 1). *Proteus mirabilis* is a flagellate bacterium that has the ability to render urine alkaline, producing magnesium and calcium phosphate microcrystals that accumulate on the catheter surface forming a mineralized biofilm structure^(6,9)^. Microcrystals adhered to the bacterium may remain viable after catheter removal and trigger chronic urinary infections, recurrent catheter lumen blockage, acting as re-infection reservoirs after UTI treatment or device replacement^(6)^. Due to its high migration capacity and because it contains elongated cells, *Proteus mirabilis* is able to easily displace from the periurethral region into the bladder which can often lead to an eventual reflux of infected urine into the upper urinary tract and kidneys^(6,9)^. In this study, 33.3% of participants showed magnesium-ammonium-phosphate in urine when viewed under optical microscopy (Figure 1) and the same samples coincided with *Proteus mirabilis* at the IBD tip.

Another bacteria prevalent at the IBD tip was *Enterococcus faecalis*, and it can be stated, according to another experimental study, that the factors secreted by *Enterococcus faecalis* increase the pathogenic potential of *Proteus mirabilis*, promoting a high urease production and increased cytotoxicity^(4)^. In this study, two samples had the co-infection of the two bacteria at the IBD tip (Chart 1), which could have increased the biofilm biomass, potentiated AC-UTI and risk of urinary focus sepsis and bacteremia, mainly due to the synergistic interaction between the two microorganisms.

In this sense, the structural analyses of the IBD visualized in SEM and of microorganisms in urine culture made it possible to infer that, for patients in this study who removed IBD, there was a possibility of presenting CA-UTI, since, associated with a positive urine culture, all of them reported minor suprapubic tenderness and costovertebral angle pain on physical examination. In addition to this, nursing must perform an active and daily surveillance of the catheter length of stay, indication and length of stay of patients with IBD, indicators established and associated with UTI-AC prevalence maintenance^(15)^.

## CONCLUSION

This research sought to broaden and give a new meaning to the view nursing team’ view on CA-UTI, since knowing the formation of biofilms in the IBD and the results of urine culture of all patients analyzed, it is understood the need for a collective assessment of the entire multidisciplinary team for a decision on strict criteria for physical and clinical examinations of all patients expected to remove the IBD and confirmation of the need to request a urine culture before discharge.

This study also contributed to generate quality indicators that contribute to the development and implementation of CA-UTI prevention and management methods, in order to avoid adverse events in patients, often unknown by the multidisciplinary team.

As already evidenced in the results, it is necessary to be attentive; therefore, the use of antibiotics, which for the most part was started entirely empirically, the growth of pathogenic microorganisms in the urine, the formation of crystalline precipitates, the development of bacterial biofilm occur and, together with this, length of hospital stay and morbidity and mortality can increase in the institution.

## ASSOCIATE EDITOR

Dulce Aparecida Barbosa
